# Artificial Intelligence–Based Electrocardiogram Model as a Predictor of Postoperative Atrial Fibrillation Following Cardiac Surgery: Retrospective Cohort Study

**DOI:** 10.2196/77164

**Published:** 2025-11-10

**Authors:** Changho Han, Sarah Soh, Je-Wook Park, Hui-Nam Pak, Dukyong Yoon

**Affiliations:** 1 Department of Biomedical Systems Informatics, Yonsei University College of Medicine, 331 Dongbaekjukjeon-daero, Giheung-gu, Yongin, 16995, Republic of Korea, 82 3151898450; 2 Department of Anesthesiology and Pain Medicine, Yonsei University College of Medicine, Seoul, Republic of Korea; 3 Institute for Innovation in Digital Healthcare, Yonsei University, Seoul, Republic of Korea; 4 Division of Cardiology, Department of Internal Medicine, Yongin Severance Hospital, Yonsei University College of Medicine, Yongin, Republic of Korea; 5 Division of Cardiology, Department of Internal Medicine, Yonsei University College of Medicine, Seoul, Republic of Korea; 6 Center for Digital Health, Yongin Severance Hospital, Yonsei University Health System, Yongin, Republic of Korea

**Keywords:** postoperative atrial fibrillation, cardiac surgery, electrocardiogram, artificial intelligence, deep learning

## Abstract

**Background:**

Postoperative atrial fibrillation (AF) after cardiac surgery is common and is associated with substantial clinical and economic repercussions. However, existing strategies for preventing postoperative AF remain suboptimal, limiting proactive management. Advances in artificial intelligence (AI) may improve the prediction of postoperative AF. Studies have shown that deep learning applied to electrocardiograms (ECGs) can detect subtle patterns in non-AF ECGs associated with a history of (or impending) AF (referred to as the AI-ECG-AF model). As a noninvasive test routinely performed throughout the perioperative period, the ECG presents a unique opportunity for additional risk stratification.

**Objective:**

We aimed to determine whether the AI-ECG-AF model can serve as an independent risk factor for postoperative AF after cardiac surgery, compare its predictive performance with existing postoperative AF prediction tools, and assess its additive value.

**Methods:**

This single-center retrospective cohort study included 2266 patients (5402 standard 12-lead ECGs) who underwent cardiac surgery at a tertiary hospital in South Korea between December 2018 and December 2023. The AI-ECG-AF model was trained on 4.05 million non-AF standard 12-lead ECGs (1.13 million patients) using a 1D EfficientNet-B0 architecture and achieved an area under the receiver operating characteristic curve (AUROC) of 0.901 (95% CI 0.900‐0.902) in its held-out test set. Postoperative AF was defined as AF documented by ECG within 30 days after surgery. Using multivariable logistic regression, we assessed the association between the AI-ECG-AF model score and postoperative AF, adjusting for conventional clinical variables. We also investigated the additive or synergistic predictive value of the AI-ECG-AF model score when combined with an existing postoperative AF tool (the postoperative atrial fibrillation score) or other risk factors, based on the AUROC.

**Results:**

After adjusting for other clinical variables, a 10% absolute increase in the AI-ECG-AF model score was associated with a 1.197- to 1.209-fold increase in the odds of developing postoperative AF. The AI-ECG-AF model score significantly enhanced postoperative AF prediction: the AUROC of the existing postoperative atrial fibrillation score was 0.643; adding the AI-ECG-AF model score increased it to 0.680 (*P*<.001), and combining the AI-ECG-AF model score with other risk factors raised it to 0.710 (*P*<.001).

**Conclusions:**

The AI-ECG-AF model serves as a novel, robust, and independent risk factor for postoperative AF following cardiac surgery and provides additive or synergistic predictive value when integrated with existing postoperative AF prediction tools or other risk factors. By capturing atrial electrophysiological vulnerability not reflected in conventional clinical scores, the AI-ECG-AF model may function as a noninvasive biomarker for preoperative risk stratification for postoperative AF prediction in cardiac surgery patients, potentially enabling targeted prophylaxis and closer monitoring during the perioperative period.

## Introduction

Postoperative atrial fibrillation (AF) is recognized as the most frequently occurring complication following cardiac surgery, with reported incidence rates reaching as high as 50% in various studies [1-4]. The development of postoperative AF is associated with substantial clinical and economic repercussions, including an increased risk of mortality, heightened rates of morbidity, extended durations of hospitalization, and significantly elevated health care costs [1-4]. Despite ongoing advancements that have led to improved overall outcomes in cardiac surgery, the reported prevalence of postoperative AF has remained largely unchanged over the past several decades [2].

Despite its high prevalence and substantial clinical impact, existing strategies for preventing postoperative AF remain suboptimal. While current guidelines endorse the perioperative administration of oral beta blockers or amiodarone to mitigate the risk of postoperative AF, surveys indicate that these medications are not routinely prescribed in clinical practice [2,5]. This reluctance is primarily driven by concerns over potential side effects, such as hypotension and bradycardia, which may introduce additional complications for surgical patients [2]. Therefore, identifying patients with a high likelihood of developing postoperative AF to initiate tailored prophylactic therapy and implement targeted monitoring during the perioperative period is crucial for effective management.

Efforts to develop or assess risk scores for predicting postoperative AF have faced challenges, as these systems have been statistically weak and inconclusive, failing to be widely accepted [6,7]. However, advances in artificial intelligence (AI) may improve postoperative AF prediction. Numerous studies have demonstrated the successful application of AI to electrocardiograms (ECGs), the electrical fingerprints of the heart [8-12]. As a noninvasive test routinely performed throughout the perioperative period, the ECG presents a unique opportunity for additional risk stratification. If postoperative AF risk could be further assessed from these widely available ECGs, it would offer significant clinical value.

Studies have shown that deep learning (ie, AI) applied to ECGs can detect subtle patterns in non-AF ECGs associated with a history of (or impending) AF (referred to as the AI-ECG-AF model) [13]. AF is often paroxysmal and asymptomatic and thus frequently goes undetected in its early stages. However, even subclinical AF represents a major risk factor for stroke, highlighting the importance of early detection. In this context, the AI-ECG-AF model has important implications for AF screening. AI-ECG-AF can be understood as a model that identifies subtle ECG patterns potentially associated with structural substrates for AF, thereby serving as a tool for detecting preexisting atrial vulnerability [13].

In patients undergoing cardiac surgery, those with underlying atrial vulnerabilities are at elevated risk of developing postoperative AF when exposed to the stressors of cardiac surgery [1,14,15]. Therefore, the AI-ECG-AF model may hold significant value in identifying individuals at increased risk of postoperative AF in this population.

In this study, we evaluated whether the AI-ECG-AF model score could serve as an independent risk factor for postoperative AF following cardiac surgery. Furthermore, we evaluated its additive or synergistic predictive value when combined with other risk factors or an existing postoperative AF prediction tool. In doing so, we explored the potential applicability of the AI-ECG-AF model as a novel biomarker for postoperative AF after cardiac surgery, leveraging its ability to detect latent atrial vulnerability. By capturing electrophysiological vulnerability that may not be reflected in conventional clinical scores, the AI-ECG-AF model may function as a noninvasive biomarker for preoperative risk stratification and improve the accuracy of existing preoperative risk prediction strategies.

## Methods

### Ethical Considerations

The Department of Anesthesiology at Severance Hospital in Seoul, South Korea, has established a cardiac surgery registry that compiles extensive perioperative clinical data. The hospital’s institutional review board approved this study (approval numbers: 4-2022-1506 and 4-2024-1515), waived the requirement for informed consent because only anonymized data were used retrospectively, and limited data usage to the approved timeframe. All data were de-identified before analysis in accordance with institutional anonymization protocols, and no personally identifiable information was accessible to the investigators. As this study analyzed retrospective anonymized registry data without direct participant involvement, no financial or other compensation was provided to participants. No images or supplementary materials contain identifiable individuals.

### Cardiac Surgery Cohort Data

**Figure 1.**
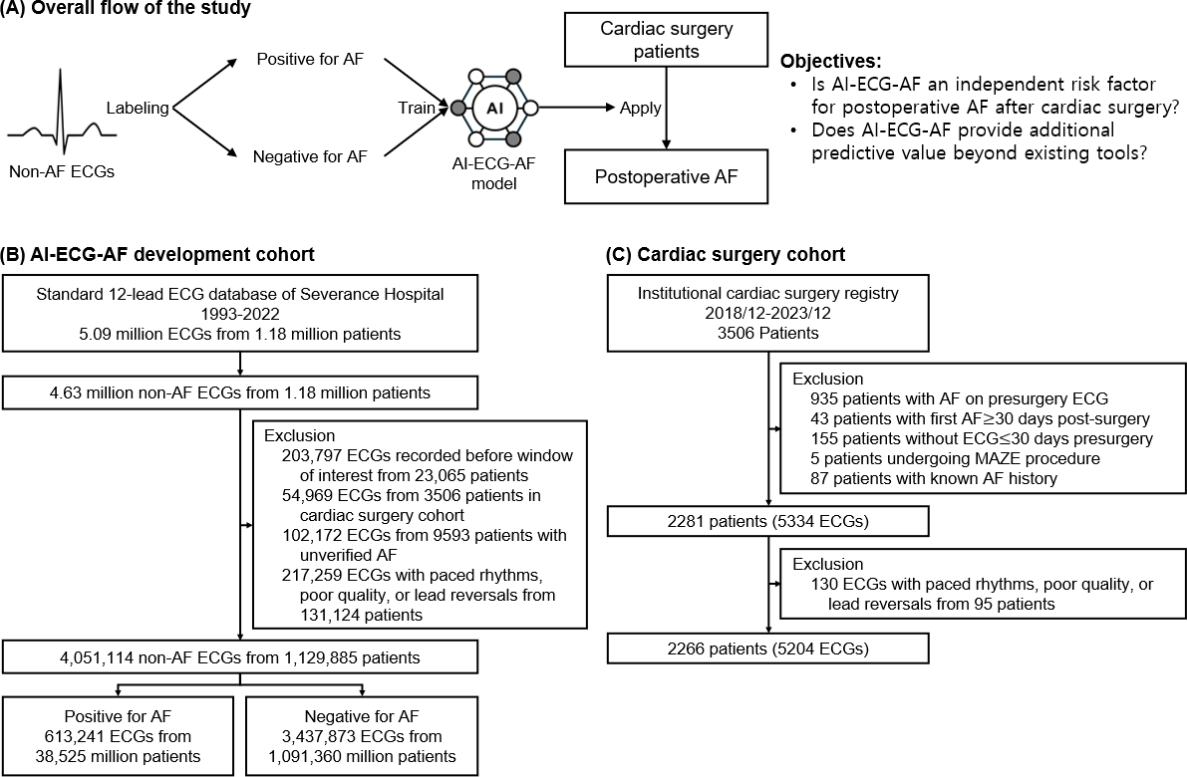
Flow of the study and the datasets. (A) The overall study flow involves labeling non-AF ECGs for that history of (or impending) AF, training the AI-ECG-AF model, and applying it to cardiac surgery patients to evaluate whether the AI-ECG-AF model score is an independent risk factor for postoperative AF and assess its additive predictive value beyond existing tools. (B) The AI-ECG-AF model development cohort was derived from a large ECG database containing 5.09 million ECGs from 1.18 million patients. For AI-ECG-AF model development, 4.05 million non-AF ECGs from 1.13 million patients were used for modeling. (C) The cardiac surgery cohort was selected from an institutional registry, with a final cohort of 5204 ECGs from 2266 patients. AF: atrial fibrillation; AI: artificial intelligence; ECG: electrocardiogram.

Adult patients (age ≥19 y) who underwent cardiac surgery at Severance Hospital between December 2018 and December 2023 were included (Figure 1). For patients who underwent multiple surgeries during this period, only the first surgery was included. Preoperative clinical data reflecting patient status, including demographics, medical history, laboratory results, physiological measurements, medications, and type of surgery were collected from the institutional cardiac surgery registry (Table S1 in Multimedia Appendix 1). Variables with missing rates exceeding 5% in the entire cohort were excluded, and the remaining missing data were handled using mean imputation.

Standard 10-second 12-lead ECGs of these patients were also extracted. AF ECGs were defined as those with an automatic interpretation indicating *atrial fibrillation* or *atrial flutter*. We excluded patients who had an AF ECG recorded before surgery, those with a known history of AF, or those undergoing the MAZE procedure (a surgical treatment for AF). For the remaining patients, only ECGs taken within 30 days before the surgery were included in the analysis. ECGs with automatic interpretations indicating paced rhythms, poor quality, or lead reversals were also excluded. While there is no consensus on the precise definition of postoperative AF [1,16,17], we defined it as AF documented by ECG within 30 days after surgery, with this 30-day cutoff being widely used across multiple studies. Patients with first AF observed after this 30-day postoperative period were excluded. A sensitivity analysis was conducted using a 7-day definition for postoperative AF.

### AI-ECG-AF Model Development

Our primary objective was to evaluate the AI-ECG-AF model from a clinical perspective, focusing on its utility as a novel predictive biomarker for postoperative AF in patients undergoing cardiac surgery. Accordingly, we developed the AI-ECG-AF model using a methodology similar to that of a previous study [13], with some modifications, and concentrated our efforts on its clinical evaluation. The details of the model development are described below.

The standard 12-lead ECG database from Severance Hospital contained 5.1 million ECGs from 1.2 million patients from 1993 to 2022 (Figure 1). The ECGs were utilized in their original form as raw 1D waveform time-series data. All non-AF ECGs were used for modeling. We classified patients into two groups: patients positive for AF, who had at least one AF ECG recorded in the database, and patients negative for AF, who had no AF ECG recorded in the database and had no diagnostic codes for AF in their electronic medical record. Patients who had an AF diagnosis code but lacked ECG confirmation were excluded from the analysis to avoid ambiguity (referred to as *unverified AF*). ECGs with automatic interpretations indicating paced rhythms, poor quality, or lead reversals were excluded. ECGs from patients included in the cardiac surgery cohort were also excluded to prevent data leakage.

For patients positive for AF, we used non-AF ECGs recorded starting from 31 days before their first AF ECG and onward. Structural changes associated with AF would be present before the first recorded AF episode, so while we could use ECGs from before the first AF ECG, we chose a relatively short time interval as a conservative measure to avoid using ECGs before any structural changes developed, as done in the previous study [13]. For patients negative for AF, all their non-AF ECGs were used for modeling.

We used the 1D EfficientNet-B0 architecture (Figure S1 in Multimedia Appendix 1) [18]. Given the extensive size of the dataset, we utilized only the middle 5 seconds of the 10-second ECG (ie, from 2.5 to 7.5 s). The ECGs, originally at 250 or 500 Hz, were standardized to 250 Hz by downsampling the 500 Hz. Each lead was z-normalized (mean=0, standard deviation=1). No additional signal filtering was applied. Since four of the six limb leads can be linearly derived from any two leads using the Einthoven law and the Goldberger equation [19,20], we used eight leads (I, II, V1-V6) as input. We randomly split the dataset at a 6:2:2 ratio (stratified) into training, validation, and test sets while ensuring no individual overlap. During each training epoch, we balanced classes by randomly oversampling the minority class and undersampling the majority class to equal sizes while maintaining the original dataset size. No such resampling was applied to the validation or test sets, which were kept in their original distributions. Hyperparameter optimization was performed through a grid search based on learning rate, batch size, and weight decay. We used the Adam optimizer, and a learning rate of 0.003, batch size of 512, and weight decay of 0 were ultimately selected. A learning rate halving strategy was applied, reducing the learning rate by half if the validation loss from the validation set did not improve for 5 consecutive epochs. The model was trained on the training set, with early stopping applied if the validation loss from the validation set did not improve for up to 15 consecutive epochs. The final evaluation was conducted on the test set. The model produces an output score ranging from 0 to 1, where higher scores indicate a greater likelihood of AF as predicted by the model.

### Objectives

Our first objective was to verify whether the AI-ECG-AF model score is an independent risk factor for postoperative AF in cardiac surgery patients. We applied the AI-ECG-AF model to the ECGs from our cardiac surgery cohort to obtain prediction outputs. We performed multivariable logistic regression with postoperative AF as the dependent variable and the AI-ECG-AF model score along with clinical variables as independent variables. To ensure the robustness of results, we conducted the logistic regression analyses with two sets of clinical variables.

In the first logistic regression, we included both the AI-ECG-AF model score and clinical variables from the POAF score [7], an existing postoperative AF prediction tool, which comprised age category (<60, ≥60 and <70, ≥70 and <80, and ≥80), chronic obstructive pulmonary disease (COPD), estimated glomerular filtration rate <15 mL/min, emergency surgery, preoperative intra-aortic balloon pump/extracorporeal membrane oxygenation/ventricular assist device, left ventricular ejection fraction <30%, and valve surgery as independent variables.

In the second logistic regression, we utilized all preoperative clinical data from the institutional cardiac surgery registry (Table S1 in Multimedia Appendix 1) and performed stepwise logistic regression with backward elimination, incorporating the AI-ECG-AF model score alongside clinical variables as independent variables. The resulting postoperative AF prediction score was termed the AI-enhanced post-cardiac-operative AF (AI-COAF) score.

Subgroup analyses by sex, age (<65 and ≥65 y), and type of surgery (valve and nonvalve) were additionally performed for both logistic regression analyses. For the second logistic regression, the subgroup analyses utilized the predetermined dependent variables identified during the backward selection process to ensure consistency.

Our second objective was to evaluate the additive or synergistic predictive value of the AI-ECG-AF model score when combined with other risk factors or the existing POAF score [7]. We compared the AI-ECG-AF’s predictive ability (via the area under the receiver operating characteristic curve [AUROC] and average precision) with the POAF score and tested if adding the AI-ECG-AF score to the POAF score (AI-POAF [artificial-intelligence–augmented postoperative atrial fibrillation] score=POAF score+α×AI-ECG-AF score) improved predictions. We found the optimal weighting factor (*α*=4) using data from December 2018 to December 2021 (derivation dataset). All model comparisons used data from January 2022 to December 2023 (comparison dataset). We also retrained the AI-POAF score using the predetermined dependent variables on the derivation dataset and compared its performance with other models on the comparison dataset. Performance metrics, including accuracy, sensitivity, specificity, positive predictive value, negative predictive value, and the *F*_1_-score, were calculated in the comparison dataset using the threshold that maximized the Youden J index in the derivation dataset.

### Statistical Analyses and Software

Normality of continuous variables was assessed with the Shapiro-Wilk test. Since no continuous variables were normally distributed, Mann-Whitney *U* tests were used for comparisons. Categorical variables were compared using the *χ*^2^ test. The determination of 95% CI of AUROC and AUROC comparisons used the Delong’s method (paired and two-sided) [21]. Statistical significance was set at *P*<.05.

Model training and evaluation were performed in Python (version 3.10.6) using Pytorch (version 2.0.1) and Scikit-learn (version 1.3.0), while statistical analyses were conducted with the TableOne package (version 0.8.0). Regression analyses were performed in R (version 4.0.4).

## Results

### Performance of the AI-ECG-AF Model

**Figure 2.**
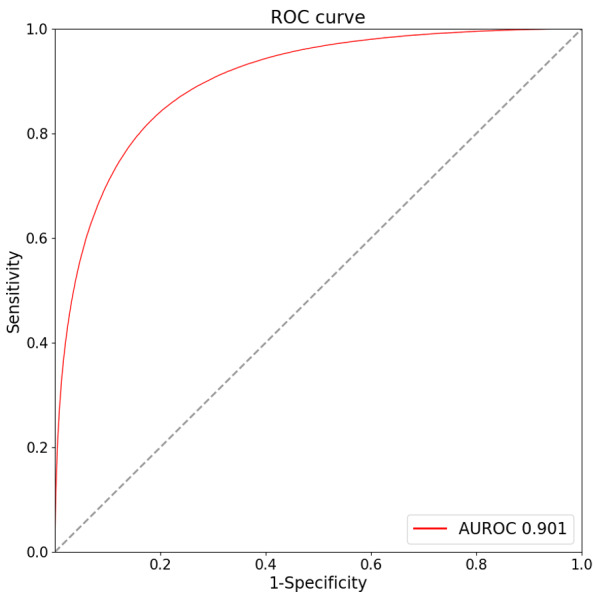
ROC curve of the AI-ECG-AF model in the test set of its development cohort. The AI-ECG-AF model achieved an AUROC of 0.901 (95% CI 0.900‐0.902) for identifying the electrocardiographic signature of AF in non-AF ECGs in the test set of the AI-ECG-AF development cohort. AF: atrial fibrillation; AI: artificial intelligence; AUROC: area under the receiver operating characteristics curve; ECG: electrocardiogram; ROC: receiver operating characteristic.

For AI-ECG-AF development, 4.05 million non-AF ECGs from 1.13 million patients were used for modeling (Figure 1). Within this dataset, a subset of 0.61 million ECGs, corresponding to 0.04 million patients, was labeled as positive for AF, while the remaining 3.45 million ECGs, derived from 1.09 million patients, were categorized as negative for AF. Among all the ECGs included in the AI-ECG-AF development process, the average age at the time of measurement was 60.3 years, with a standard deviation of 10.3 years. Additionally, 55.3% of the ECGs in the dataset were recorded from male patients. In terms of performance, the AI-ECG-AF model achieved an AUROC of 0.901, with a 95% CI ranging from 0.900 to 0.902, for identifying the electrocardiographic signature of AF in non-AF ECGs in the test set of the AI-ECG-AF development cohort (Figure 2). The fully developed AI-ECG-AF model, along with comprehensive instructions detailing its usage, has been made entirely accessible to the public through our dedicated online repository [22].

### Dataset Characteristics

A total of 5204 ECGs obtained from a cohort of 2266 patients who had undergone cardiac surgery were included in the final analysis (Table 1 and Figure 1). Patients with postoperative AF (1815 ECGs from 763 patients) had a lower proportion of males (66.7% [1210/1815] vs 70.5% [2390/3389], *P*=.005), were older (≥80 y: 8.8% [159/1815] vs 3.2% [109/3389]; 70‐79 y: 40.1% [727/1815] vs 27.1% [919/3389]; *P*<.001), had a higher prevalence of COPD (7.6% [138/1815] vs 3.3% [112/3389], *P*<.001), a higher rate of valve surgery (47.7% [865/1815] vs 41.5% [1405/3389], *P*<.001), and higher POAF score (median [IQR]: 2 [1-3] vs 2 [1-2], *P*<.001), and AI-ECG-AF model scores (median [IQR]: 0.553 [0.313‐0.763] vs 0.346 [0.154‐0.601], *P*<.001) compared to those who were negative for postoperative AF (3389 ECGs from 1503 patients). The remaining preoperative clinical characteristics from the institutional cardiac surgery registry are summarized in Table S2 in Multimedia Appendix 1. Figure S2 in Multimedia Appendix 1 provides a visual representation of the incidence of POAF across different postoperative days, showing that the highest incidence occurred between postoperative days 1 and 4.

**Table 1.** Patient characteristics.

	Postoperative AF^a^ negative (n=3389)	Postoperative AF positive (n=1815)	*P* value
Number of patients, n	1503	763	
Sex (male), n (%)	2390 (70.5)	1210 (66.7)	.005
Age category, n (%)			<.001
<60 years	1269 (37.4)	318 (17.5)	
60 to 69 years	1092 (32.2)	611 (33.7)	
70 to 79 years	919 (27.1)	727 (40.1)	
80 years	109 (3.2)	159 (8.8)	
COPD^b^, n (%)	112 (3.3)	138 (7.6)	<.001
eGFR^c^ <15 mL/min, n (%)	203 (6.)	130 (7.2)	.112
Emergency surgery, n (%)	108 (3.2)	49 (2.7)	.371
Preoperative IABP^d^/ECMO^e^/VAD^f^, n (%)	43 (1.3)	28 (1.5)	.492
LVEF^g^ <30%, n (%)	241 (7.1)	150 (8.3)	.147
Valve surgery, n (%)	1405 (41.5)	865 (47.7)	<.001
POAF^h^ score, median [IQR^i^]	2 [1 – 2]	2 [1 – 3]	<.001
AI^j^-ECG^k^-AF^a^ model score, median [IQR]	0.346 [0.154‐0.601]	0.553 [0.313‐0.763]	<.001

a AF: atrial fibrillation.

b COPD: chronic obstructive pulmonary disease.

c eGFR: estimated glomerular filtration rate.

d IABP: intra-aortic balloon pump.

e ECMO: extracorporeal membrane oxygenation.

f VAD: ventricular assist device.

g LVEF: left ventricular ejection fraction.

h POAF: postoperative atrial fibrillation.

i IQR: interquartile range.

j AI: artificial intelligence.

k ECG: electrocardiogram.

### Regression Analyses Results

**Figure 3.**
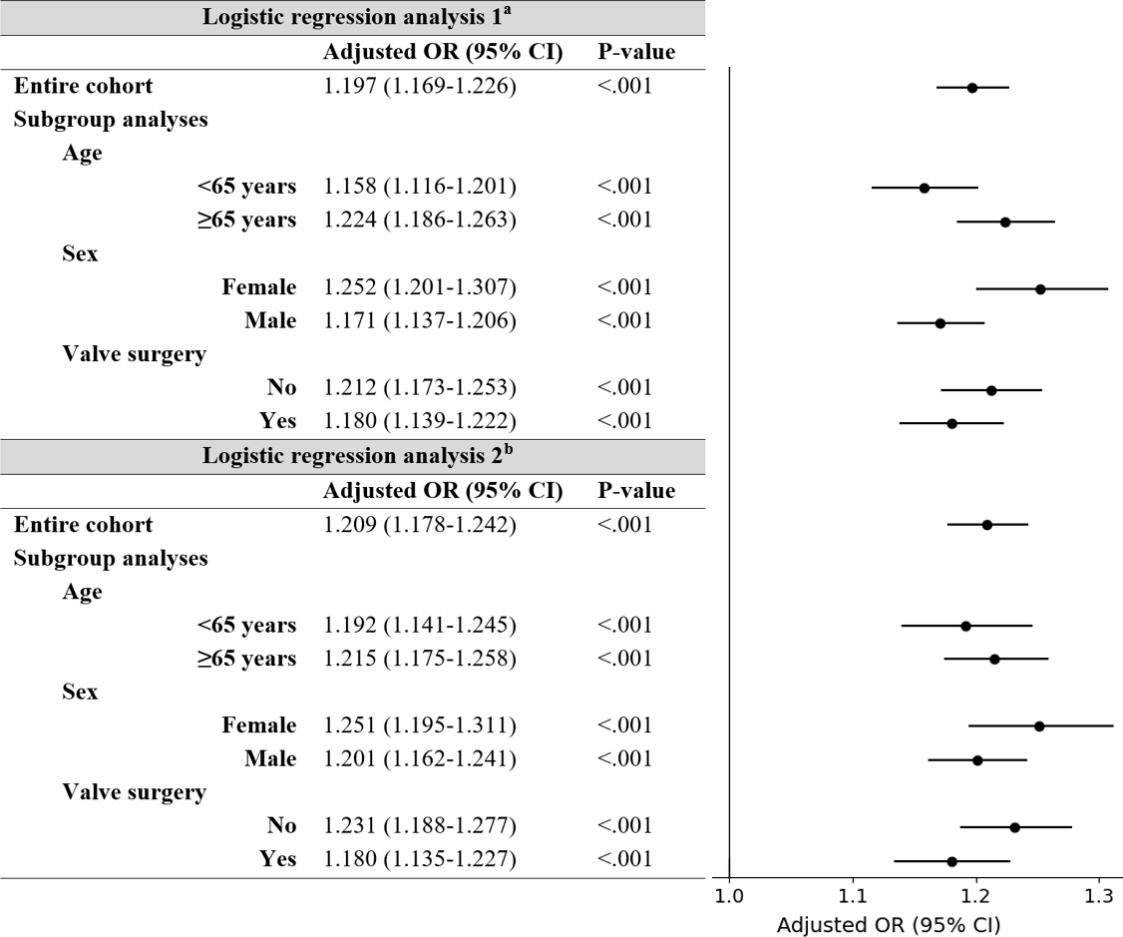
Adjusted odds ratios per 10% absolute increase in AI-ECG-AF model score. To facilitate a more intuitive interpretation, the AI-ECG-AF model scores, originally on a scale from 0 to 1, were rescaled to a range of 0 to 10 by multiplying by ten. As a result, the odds ratios for the AI-ECG-AF model scores now reflect the change in odds associated with a 10% absolute increase in the AI-ECG-AF model score after adjusting for other clinical variables. Logistic regression analysis 1 was adjusted for variables included in the POAF score. Logistic regression analysis 2 was adjusted for variables extracted from the institutional cardiac surgery registry, as detailed in Table S1 in Multimedia Appendix 1, with variables selected using stepwise logistic regression with backward elimination. OR: odds ratio; POAF: postoperative atrial fibrillation.

Figure 3 shows the results of the logistic regression analyses. To facilitate a more intuitive interpretation, the AI-ECG-AF model scores, originally on a scale from 0 to 1, were rescaled to a range of 0 to 10 by multiplying by ten. This adjustment allows the odds ratios displayed in Figure 3 to represent the change in odds associated with a 10% absolute increase in the AI-ECG-AF model score after accounting for other relevant clinical variables.

After adjusting for the variables included in the POAF score, a 10% absolute increase in the AI-ECG-AF model score was found to be associated with a 19.7% rise in the odds of developing postoperative AF (95% CI 16.9%‐22.6%). The AI-ECG-AF model score was identified as an independent risk factor for postoperative AF, not only when analyzing the entire patient cohort but also consistently across all demographic and surgical subgroups. A comprehensive list of odds ratios for all analyzed variables is provided in Table S3 in Multimedia Appendix 1.

Similarly, after adjusting for variables extracted from the institutional cardiac surgery registry, which were selected using stepwise logistic regression with backward elimination, a 10% absolute increase in the AI-ECG-AF model score was associated with a 20.9% rise in the odds of developing postoperative AF (95% CI 17.8%‐24.2%). The AI-ECG-AF model score remained an independent risk factor for postoperative AF in the entire cohort as well as across all demographic and surgical subgroups. The odds ratios for all variables are provided in Table S4 in Multimedia Appendix 1.

To further assess the robustness of these findings, a sensitivity analysis was performed using a more stringent 7-day definition for postoperative AF. In this analysis, an additional 188 ECGs from 72 patients, who had their first recorded AF episode occurring beyond the initial 7-day postoperative period but within 30 days, were excluded. The AI-ECG-AF model score remained a significant independent risk factor for postoperative AF across both regression models, with adjusted odds ratios for AI-ECG-AF model score×10 of 1.200 (95% CI 1.171‐1.231) and 1.221 (95% CI 1.188‐1.254), respectively (Figure S3 in Multimedia Appendix 1). Furthermore, the association remained consistent across all demographic and surgical subgroups.

### Predictive Performance for Postoperative Atrial Fibrillation

**Figure 4.**
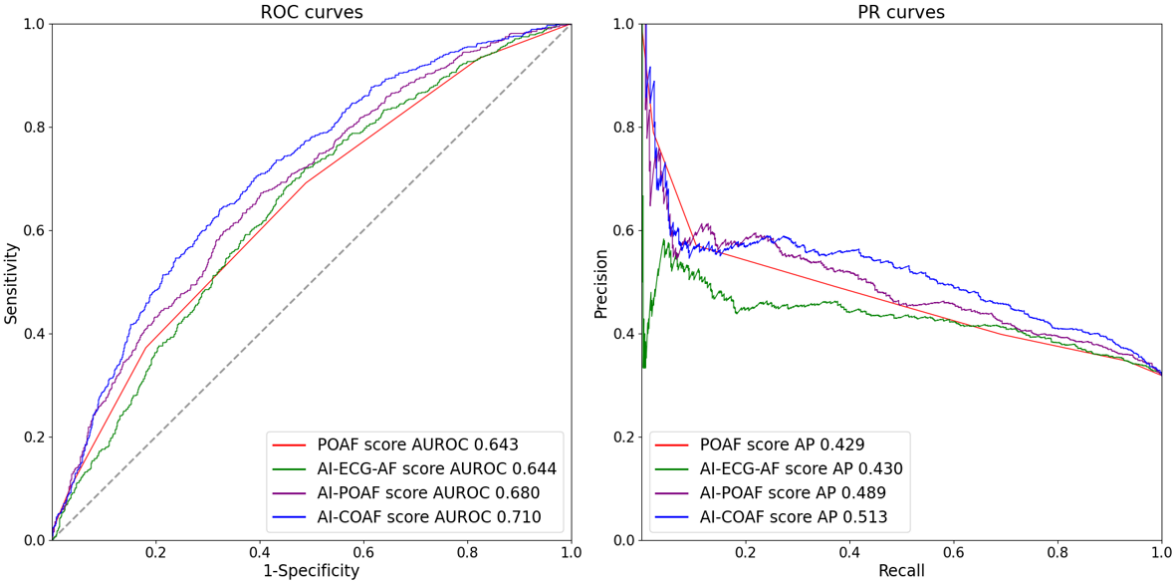
ROC and PR curves of the models in the comparison dataset. AUROC comparisons: POAF score versus AI-ECG-AF model score (*P*=.927), POAF score versus AI-POAF score (*P*<.001), and POAF score versus AI-COAF score (*P*<.001). AF: atrial fibrillation; AI: artificial intelligence; AI-COAF score: AI-enhanced post-cardiac-operative AF score; AP: average precision; AUROC: area under the receiver operating characteristic curve; ECG: electrocardiogram; POAF: postoperative atrial fibrillation; PR: precision-recall; ROC: receiver operating characteristic.

Figure 4 shows the ROC and precision–recall curves of the models for predicting postoperative AF. The AUROCs were 0.643, 0.644, 0.680, and 0.710 for the POAF score, AI-ECG-AF model score, AI-POAF score, and AI-COAF score, respectively. According to the Delong test, both the AI-POAF score and AI-COAF score demonstrated significantly higher AUROCs than the POAF score (both *P*<.001), whereas there was no statistically significant difference between the POAF score and the AI-ECG-AF model score (*P*=.927). The respective average precisions were 0.429, 0.430, 0.489, and 0.513. Table 2 shows the performance at the threshold determined at maximum Youden J index.

**Table 2.** Performance of the models at maximum Youden J index.

	Accuracy	Sensitivity	Specificity	PPV^a^	NPV^b^	*F*_1_-score
POAF^c^ score	0.569	0.692	0.511	0.398	0.780	0.506
AI^d^-ECG^e^-AF^f^ model score	0.582	0.701	0.526	0.409	0.790	0.517
AI-POAF^g^ score	0.649	0.557	0.693	0.459	0.770	0.503
AI-COAF score	0.663	0.646	0.671	0.479	0.802	0.550

a PPV: positive predictive value.

b NPV: negative predictive value.

c POAF: postoperative atrial fibrillation.

d AI: artificial intelligence.

e ECG: electrocardiogram.

f AF: atrial fibrillation.

g AI-COAF score: AI-enhanced post-cardiac-operative AF score.

For the sensitivity analysis using a 7-day definition for postoperative AF, the AUROCs were 0.647, 0.641, 0.678, and 0.706 for the POAF score, AI-ECG-AF model score, AI-POAF score, and AI-COAF score, respectively (Figure S4 in Multimedia Appendix 1). Consistent with the primary analysis, the Delong test indicated that the AUROCs of the AI-POAF score and AI-COAF score remained significantly higher than that of the POAF score (both *P*<.001), while no significant difference was observed between the POAF score and the AI-ECG-AF model score (*P*=.692).

## Discussion

### Principal Findings

In this study, we evaluated the potential of the AI-ECG-AF model as a predictor for postoperative AF in cardiac surgery patients. After adjusting for other clinical variables, a 10% absolute increase in the AI-ECG-AF model score was associated with a 1.197- to 1.209-fold increase in the odds of developing postoperative AF. We demonstrated that the AI-ECG-AF model score provides additive or synergistic predictive value when integrated with an existing postoperative AF prediction tool or other risk factors: the AUROC of the existing POAF score was 0.643; adding the AI-ECG-AF model score increased it to 0.680 (*P*<.001), and combining the AI-ECG-AF model score with other risk factors raised it to 0.710 (*P*<.001).

### Comparison to Prior Work

Attempts to predict postoperative AF have faced significant challenges. Various studies have evaluated various scoring systems, originally developed for different purposes, for predicting postoperative AF in cardiac surgery patients [6,23]. For example, the European System for Cardiac Operative Risk Evaluation (for predicting mortality after cardiac surgery) [24], the Congestive Heart Failure, Hypertension, Age≥75 years, Diabetes Mellitus, Stroke–Vascular Disease, Age 65-74 years, and Sex Category Score (for predicting stroke risk in AF patients) [25], and the Hypertension, Age≥75 years, Transient Ischemic Attack or Stroke, Chronic Obstructive Pulmonary Disease, and Heart Failure Score (for predicting progression from paroxysmal to sustained AF) [26] have been evaluated for predicting postoperative AF in cardiac surgery patients. However, these scoring systems have only demonstrated moderate performance at best [6,23]. Although the POAF score was specifically developed for predicting postoperative AF in cardiac surgery patients [7], it similarly showed only moderate performance and has not been widely adopted in clinical practice or subsequent research [6,23]. There remains a clear unmet need for more effective postoperative AF risk stratification in cardiac surgery patients, and modern approaches such as AI may offer better solutions.

### Clinical Implications

AI applications to ECG, the electrical fingerprint of the heart, are opening up new possibilities. There is growing evidence that advanced AI techniques with deep convolutional neural networks are capable of detecting subtle signals and patterns from ECGs that do not fit traditional knowledge and are unrecognizable by the human eye, enabling prediction of diseases that were previously unpredictable with ECGs [8-12,27,28]. The AI-ECG-AF model exemplifies this advancement, as it detects the electrocardiographic signature of AF present in non-AF ECGs [13]. This has particularly important implications because AF is often paroxysmal and therefore difficult to capture during ECG screening, yet it remains a major risk factor for stroke that requires anticoagulation [13,29]. There is robust evidence supporting the real-world effectiveness of this model, with a prospective trial demonstrating that AI-ECG-AF model-guided targeted screening of AF with ECGs resulted in a significant increase in AF detection rates, particularly among those classified as high risk by the model [30].

AI-ECG-AF can be understood as a model that identifies subtle ECG patterns potentially associated with structural substrates for AF, such as atrial fibrotic changes related to aging [13,31]. Cardiac surgery induces significant stress on the heart, including inflammation, fluid shifts, and changes in the autonomic nervous system, all of which can further exacerbate preexisting atrial vulnerabilities and increase the likelihood of developing postoperative AF [3,14,15,32]. The findings from our study suggest that the AI-ECG-AF model is capable of identifying these underlying predispositions in patients undergoing cardiac surgery, allowing for the recognition of individuals at heightened risk for postoperative AF.

We demonstrated that the AI-ECG-AF model was an independent risk factor for postoperative AF and enhanced the predictive performance of conventional clinical scores. Unlike existing tools such as the POAF score—which are derived from clinical risk factors—the AI-ECG-AF model offers complementary information by capturing atrial electrophysiological vulnerability embedded in the ECG. When used in combination, the two scores provided significantly improved prediction, underscoring the potential of the AI-ECG-AF score as a novel biomarker that complements traditional clinical models and supports the implementation of more targeted prophylactic interventions and perioperative monitoring strategies. Notably, the AI-ECG-AF model, though originally developed in a general patient population, demonstrated clinical utility as a predictive biomarker even in the high-risk setting of cardiac surgery, highlighting its potential generalizability. This suggests its broader applicability, including possible integration into preoperative risk assessment protocols across various cardiac conditions. Of note, our results remained consistent irrespective of whether the broader 30-day definition or the more restrictive 7-day definition for postoperative AF was applied.

In contrast to the previous study that limited its AI-ECG-AF model development to normal sinus rhythm ECGs [13], our study incorporated all non-AF ECGs, excluding only those with lead misplacements, artifacts, or artificial pacemakers. We successfully demonstrated that the AI-ECG-AF model could maintain high performance even when processing diverse, heterogeneous ECGs, thus expanding its clinical applicability. Our model was trained on a comprehensive dataset of 4.05 million ECGs from 1.13 million patients, including 0.61 million AF-positive ECGs from 0.04 million patients. This extensive dataset helps minimize overfitting, accounts for broader ECG variability, and enhances the model’s generalizability in real-world clinical applications [33]. The developed AI-ECG-AF model, along with detailed usage instructions, has been made fully accessible to the public through our online repository [22].

Although multiple studies have demonstrated that amiodarone or beta-blockers can provide a protective effect against postoperative AF following cardiac surgery [34,35], there remains a lack of conclusive evidence supporting the routine use of these medications in all patients [36]. Furthermore, patients who develop postoperative AF are often advised to undergo long-term oral anticoagulation therapy, which has been shown to offer potential benefits in reducing the risk of thromboembolic events [37,38]. Given these considerations, accurately identifying individuals at an elevated risk of developing postoperative AF is of critical importance, as these patients require close rhythm monitoring not only in the immediate postoperative period but also throughout long-term follow-up. The AI-ECG-AF model may serve as a novel biomarker in recognizing high-risk patients who could potentially benefit from preoperative administration of amiodarone or beta-blockers, in addition to targeted strategies for the prevention of thromboembolic complications. Future studies should also aim to define and validate clinically meaningful thresholds for the AI-based scores evaluated in this study (ie, the AI-ECG-AF, AI-POAF, and AI-COAF scores), incorporating health-economic analyses and expert consensus to balance the benefits of intervention against potential harms, thereby facilitating clinical implementation.

### Limitations and Future Directions

This study’s findings should be interpreted in light of the following limitations. The study is subject to limitations inherent in its single-center retrospective design. First, as the data were derived from a single institution, the generalizability of our results to other populations or health care settings may be limited. External validation across diverse institutions is warranted to confirm the robustness of our findings. AI-ECG-AF has the advantage of being applicable using ECGs alone, but subtle waveform variations between different ECG machines may affect model performance, potentially necessitating institution-specific fine-tuning. Additionally, differences in population characteristics may require local calibration of composite scores such as AI-POAF or AI-COAF to ensure predictive accuracy. Second, our analysis was limited to cardiac surgery, yet approximately 85% of surgeries are noncardiac, which still show postoperative AF rates of 2%‐30% [39,40]. Although the mechanisms underlying POAF after noncardiac surgery are multifactorial and not fully defined, systemic inflammation, perioperative sympathetic surges, and other stressors may exacerbate atrial vulnerability and trigger AF [39,40]. Accordingly, AI-ECG-AF may also serve as a risk factor for POAF in this broader surgical population, warranting future investigation. Third, AF was defined based solely on documented episodes in stored standard 12-lead ECGs, so transient AF episodes could have been missed. In particular, AF episodes not recorded in the database—such as those detected only on continuous telemetry monitoring—or paroxysmal AF episodes occurring transiently in daily life may not have been captured. Such omissions could have influenced both the performance of the AI-ECG-AF model and the evaluation of postoperative AF. Fourth, while perioperative antiarrhythmic medications likely influence postoperative AF, we could not adjust for all such medications due to the retrospective nature of the study and the heterogeneity in medication protocols. Some antiarrhythmic medications, such as preoperative beta-blockers, were systematically recorded in our institutional registry and included in certain analyses. However, comprehensive data on all antiarrhythmic drugs were unavailable, and accurate retrospective collection was infeasible. Furthermore, detailed information such as dosage, timing, and route of administration was not reliably captured. Fifth, although AI-ECG-AF was first introduced several years ago [13], our model development did not pursue novel advances in architecture or feature extraction. Instead, we focused on clinical evaluation, assessing its utility as a predictive biomarker for POAF in cardiac surgery patients. Despite using an older architecture (EfficientNet-B0) and a standard pipeline, the model achieved an AUROC exceeding 0.9 in the development test set—deemed sufficient for clinical validation. Future studies could explore more advanced architectures or innovative techniques to enhance model performance, potentially leading to even stronger results in clinical validation. Sixth, our logistic regression analyses included multiple ECGs per patient, but we treated each as an independent observation, which may have led to underestimated standard errors and overstated statistical significance. Statistical techniques that explicitly account for within-patient correlation were not applied. Nonetheless, the large sample size and consistent subgroup findings support the robustness of our results. Finally, the AI-ECG-AF model was not trained to distinguish between different AF phenotypes (eg, paroxysmal vs persistent), and future models may be improved by incorporating more granular AF phenotype data.

### Conclusions

In conclusion, the AI-ECG-AF model serves as a novel, robust, and independent risk factor for postoperative AF following cardiac surgery and provides additive or synergistic predictive value when integrated with existing postoperative AF prediction tools or with other risk factors. By capturing atrial electrophysiological vulnerability not reflected in conventional clinical scores, the AI-ECG-AF model may function as a noninvasive biomarker for preoperative risk stratification for postoperative AF prediction in cardiac surgery patients, potentially enabling health care providers to initiate tailored prophylactic therapy and implement targeted monitoring during the perioperative period.

## Supplementary material

10.2196/77164 Multimedia Appendix 1 EfficientNet-B0 architecture, preoperative clinical data, and results of logistic regression.

## Abbreviations

AF: atrial fibrillation
AI: artificial intelligence
AI-COAF: artificial-intelligence–enhanced post-cardiac-operative atrial fibrillation score
AI-POAF: artificial-intelligence–augmented postoperative atrial fibrillation score
AUROC: area under the receiver operating characteristic curve
COPD: chronic obstructive pulmonary disease
ECG: electrocardiogram
POAF: postoperative atrial fibrillation (score)
ROC: receiver operating characteristic (curve)

## References

[1] Gaudino M, Di Franco A, Rong LQ, Piccini J, Mack M (2023). Postoperative atrial fibrillation: from mechanisms to treatment. Eur Heart J.

[2] Muehlschlegel JD, Burrage PS, Ngai JY (2019). Society of Cardiovascular Anesthesiologists/European Association of Cardiothoracic Anaesthetists Practice Advisory for the management of perioperative atrial fibrillation in patients undergoing cardiac surgery. Anesth Analg.

[3] Dobrev D, Aguilar M, Heijman J, Guichard JB, Nattel S (2019). Postoperative atrial fibrillation: mechanisms, manifestations and management. Nat Rev Cardiol.

[4] Suero OR, Ali AK, Barron LR, Segar MW, Moon MR, Chatterjee S (2024). Postoperative atrial fibrillation (POAF) after cardiac surgery: clinical practice review. J Thorac Dis.

[5] Jeppsson A, Authors/Task Force Members, (Co-Chairperson) (Sweden) (2024). 2024 EACTS Guidelines on perioperative medication in adult cardiac surgery. Eur J Cardiothorac Surg.

[6] Pandey A, Okaj I, Ichhpuniani S (2023). Risk scores for prediction of postoperative atrial fibrillation after cardiac surgery: a systematic review and meta-analysis. Am J Cardiol.

[7] Mariscalco G, Biancari F, Zanobini M (2014). Bedside tool for predicting the risk of postoperative atrial fibrillation after cardiac surgery: the POAF score. J Am Heart Assoc.

[8] Siontis KC, Noseworthy PA, Attia ZI, Friedman PA (2021). Artificial intelligence-enhanced electrocardiography in cardiovascular disease management. Nat Rev Cardiol.

[9] Attia ZI, Harmon DM, Behr ER, Friedman PA (2021). Application of artificial intelligence to the electrocardiogram. Eur Heart J.

[10] Cho Y, Yoon M, Kim J (2024). Artificial intelligence-based electrocardiographic biomarker for outcome prediction in patients with acute heart failure: prospective cohort study. J Med Internet Res.

[11] Lu SC, Chen GY, Liu AS (2025). Deep learning-based electrocardiogram model (EIANet) to predict emergency department cardiac arrest: development and external validation study. J Med Internet Res.

[12] Han C, Song Y, Lim HS (2021). Automated detection of acute myocardial infarction using asynchronous electrocardiogram signals-preview of implementing artificial intelligence with multichannel electrocardiographs obtained from smartwatches: retrospective study. J Med Internet Res.

[13] Attia ZI, Noseworthy PA, Lopez-Jimenez F (2019). An artificial intelligence-enabled ECG algorithm for the identification of patients with atrial fibrillation during sinus rhythm: a retrospective analysis of outcome prediction. Lancet.

[14] Echahidi N, Pibarot P, O’Hara G, Mathieu P (2008). Mechanisms, prevention, and treatment of atrial fibrillation after cardiac surgery. J Am Coll Cardiol.

[15] Bidar E, Bramer S, Maesen B, Maessen JG, Schotten U (2013). Post-operative atrial fibrillation - pathophysiology, treatment and prevention. J Atr Fibrillation.

[16] Meenashi Sundaram D, Vasavada AM, Ravindra C, Rengan V, Meenashi Sundaram P (2023). The management of postoperative atrial fibrillation (POAF): a systematic review. Cureus.

[17] Perezgrovas-Olaria R, Alzghari T, Rahouma M (2023). Differences in postoperative atrial fibrillation incidence and outcomes after cardiac surgery according to assessment method and definition: a systematic review and meta-analysis. J Am Heart Assoc.

[18] Tan M, Le QV (2019). EfficientNet: rethinking model scaling for convolutional neural networks. arXiv.

[19] Einthoven W (1908). Weiteres über das Elektrokardiogramm. Pflüger, Arch.

[20] Goldberger E (1942). A simple, indifferent, electrocardiographic electrode of zero potential and a technique of obtaining augmented, unipolar, extremity leads. Am Heart J.

[21] DeLong ER, DeLong DM, Clarke-Pearson DL (1988). Comparing the areas under two or more correlated receiver operating characteristic curves: a nonparametric approach. Biometrics.

[22] Changho8142/AI_ECG_AF_Yonsei.

[23] McIntyre WF (2023). Post-operative atrial fibrillation after cardiac surgery: challenges throughout the patient journey. Front Cardiovasc Med.

[24] Nashef SAM, Roques F, Sharples LD (2012). EuroSCORE II. Eur J Cardiothorac Surg.

[25] Lip GYH, Nieuwlaat R, Pisters R, Lane DA, Crijns H (2010). Refining clinical risk stratification for predicting stroke and thromboembolism in atrial fibrillation using a novel risk factor-based approach: the euro heart survey on atrial fibrillation. Chest.

[26] de Vos CB, Pisters R, Nieuwlaat R (2010). Progression from paroxysmal to persistent atrial fibrillation clinical correlates and prognosis. J Am Coll Cardiol.

[27] Yoon D, Jang JH, Choi BJ, Kim TY, Han CH (2020). Discovering hidden information in biosignals from patients using artificial intelligence. Korean J Anesthesiol.

[28] Petmezas G, Stefanopoulos L, Kilintzis V (2022). State-of-the-art deep learning methods on electrocardiogram data: systematic review. JMIR Med Inform.

[29] Joglar JA, Chung MK, Armbruster AL (2024). 2023 ACC/AHA/ACCP/HRS Guideline for the diagnosis and management of atrial fibrillation: a report of the American College of Cardiology/American Heart Association Joint Committee on clinical practice guidelines. Circulation.

[30] Noseworthy PA, Attia ZI, Behnken EM (2022). Artificial intelligence-guided screening for atrial fibrillation using electrocardiogram during sinus rhythm: a prospective non-randomised interventional trial. Lancet.

[31] Christopoulos G, Attia ZI, Van Houten HK (2022). Artificial intelligence-electrocardiography to detect atrial fibrillation: trend of probability before and after the first episode. Eur Heart J Digit Health.

[32] Bessissow A, Khan J, Devereaux PJ, Alvarez-Garcia J, Alonso-Coello P (2015). Postoperative atrial fibrillation in non-cardiac and cardiac surgery: an overview. J Thromb Haemost.

[33] Ying X (2019). An overview of overfitting and its solutions. J Phys: Conf Ser.

[34] Ozaydin M, Icli A, Yucel H (2013). Metoprolol vs. carvedilol or carvedilol plus N-acetyl cysteine on post-operative atrial fibrillation: a randomized, double-blind, placebo-controlled study. Eur Heart J.

[35] Tisdale JE, Wroblewski HA, Wall DS (2009). A randomized trial evaluating amiodarone for prevention of atrial fibrillation after pulmonary resection. Ann Thorac Surg.

[36] Van Gelder IC, Rienstra M, Bunting KV (2024). 2024 ESC Guidelines for the management of atrial fibrillation developed in collaboration with the European Association for Cardio-Thoracic Surgery (EACTS). Eur Heart J.

[37] Fragão-Marques M, Teixeira F, Mancio J (2021). Impact of oral anticoagulation therapy on postoperative atrial fibrillation outcomes: a systematic review and meta-analysis. Thromb J.

[38] Neves IA, Magalhães A, Lima da Silva G (2022). Anticoagulation therapy in patients with post-operative atrial fibrillation: systematic review with meta-analysis. Vascul Pharmacol.

[39] Joshi KK, Tiru M, Chin T, Fox MT, Stefan MS (2015). Postoperative atrial fibrillation in patients undergoing non-cardiac non-thoracic surgery: a practical approach for the hospitalist. Hosp Pract (1995).

[40] Jiang S, Liao X, Chen Y, Li B (2023). Exploring postoperative atrial fibrillation after non-cardiac surgery: mechanisms, risk factors, and prevention strategies. Front Cardiovasc Med.

[41] GitHub. CMI-Laboratory/AI_ECG_AF_POAF.

