# Supplementary material for: Artificial Intelligence–Based Electrocardiogram Model as a Predictor of Postoperative Atrial Fibrillation Following Cardiac Surgery: Retrospective Cohort Study
Source: J Med Internet Res. 2025 Nov 10;27:e77164. doi: 10.2196/77164 (PMC12603327; doi:10.2196/77164)
Supplement: Multimedia Appendix 1 [file jmir-v27-e77164-s001.docx]

**Multimedia Appendix 1: Supplementary Materials**

**Table of Contents**

**Supplementary Figures**

Figure S1. EfficientNet-B0 architecture used for AI-ECG-AF model development.

Figure S2. POAF incidence by days after cardiac surgery.

Figure S3. Adjusted odds ratios of AI-ECG-AF score from logistic regression analyses (using a 7-day definition for postoperative AF).

Figure S4. ROC and PR curves of the models in the comparison dataset, based on a sensitivity analysis using a 7-day definition for postoperative AF.

**Supplementary Tables**

Table S1. Preoperative clinical variables extracted from the institutional cardiac surgery registry.

Table S2. Additional patient characteristics.

Table S3. Results of logistic regression 1.

Table S4. Results of logistic regression 2.

**Supplementary Figures**


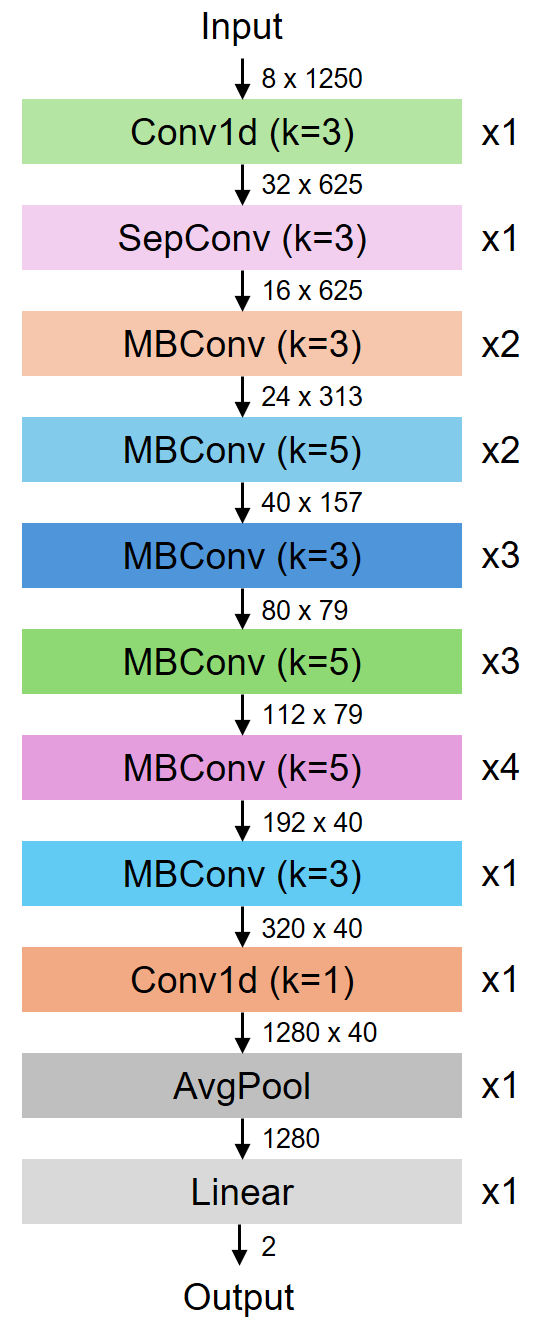


**Figure S1. EfficientNet-B0 architecture used for AI-ECG-AF model development.**

AI: artificial intelligence; ECG: electrocardiogram; AF: atrial fibrillation; Conv1d: 1-dimensional convolution; SepConv: depthwise separable convolution; MBConv: mobile inverted bottleneck convolution; AvgPool: average pooling.


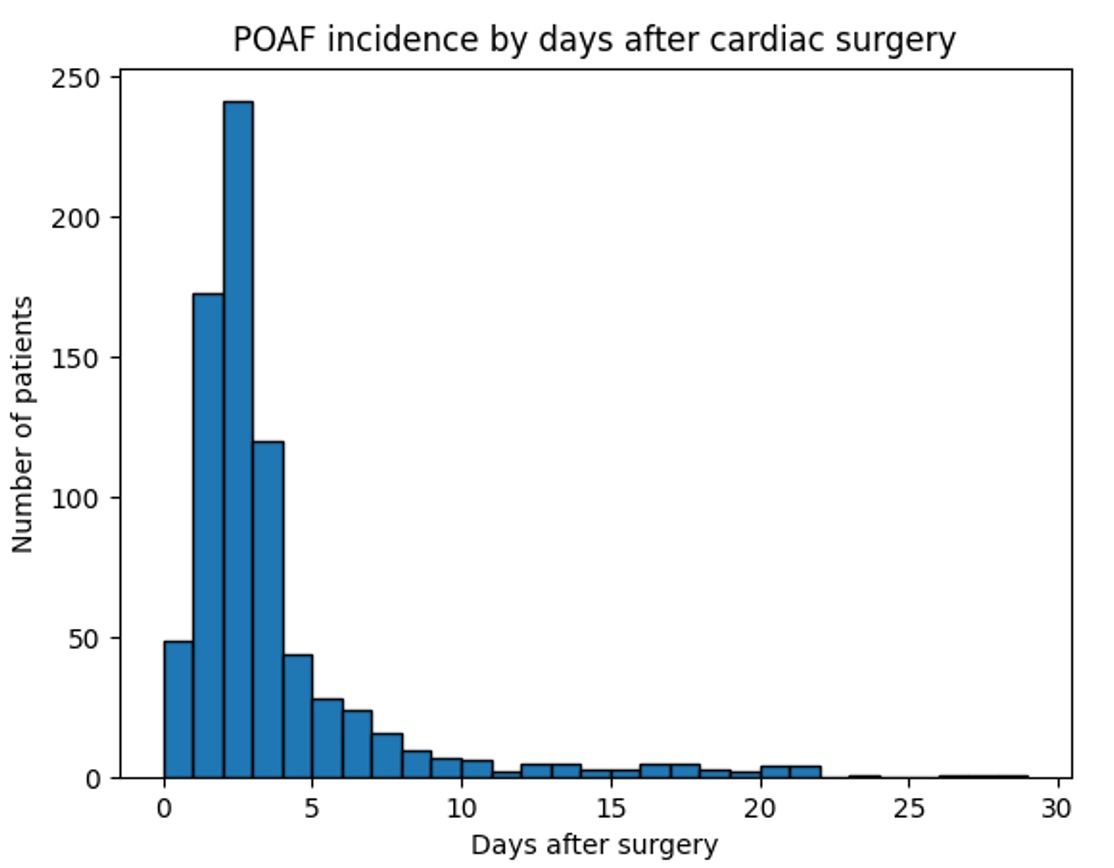


**Figure S2. POAF incidence by days after cardiac surgery.** Among the 2,266 patients included in the analysis, 763 developed POAF following cardiac surgery. The peak incidence occurred 1 to 4 days after surgery.

POAF: postoperative atrial fibrillation.

**
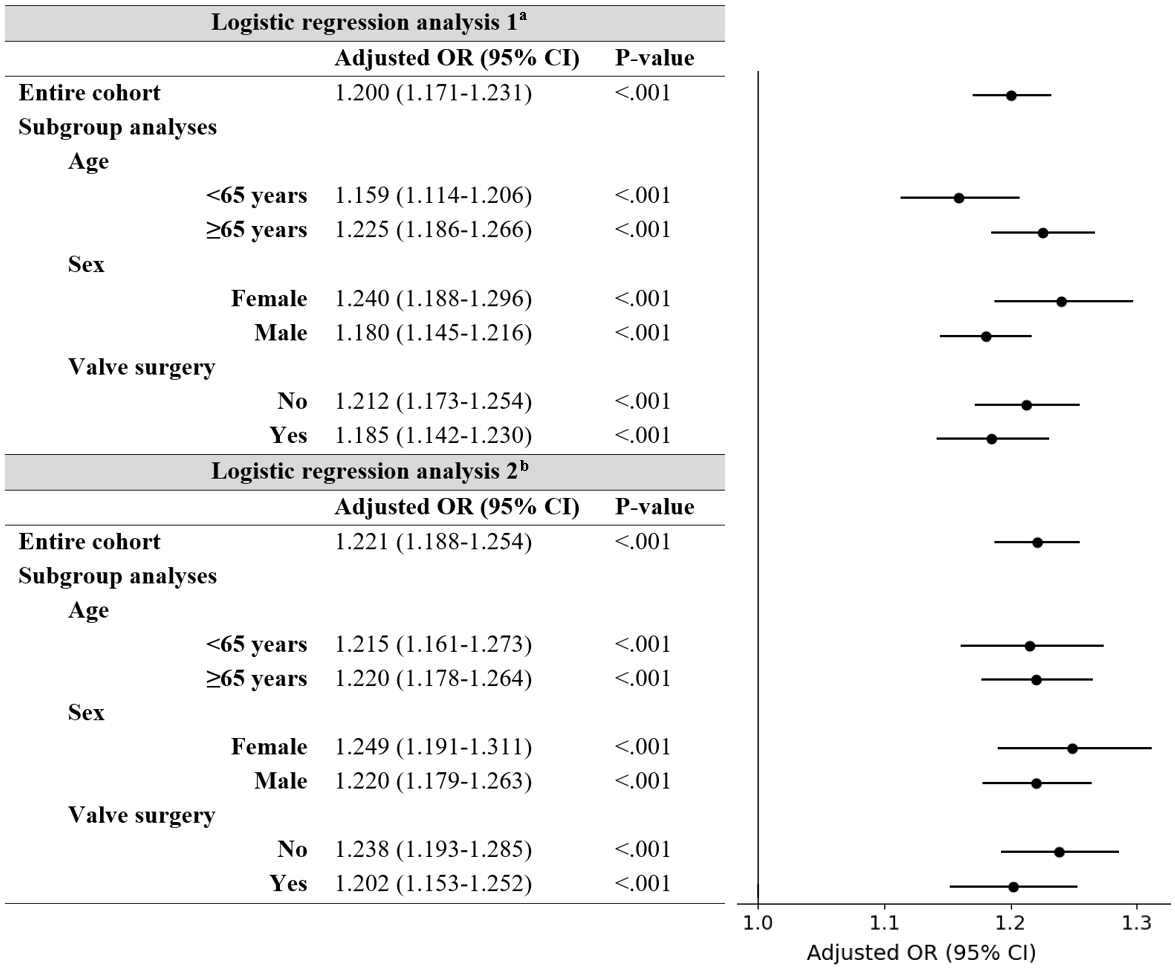
**

**Figure S3. Adjusted odds ratios per 10% absolute increase in AI-ECG-AF model score (using a 7-day definition for postoperative AF).** To facilitate a more intuitive interpretation, the AI-ECG-AF model scores, originally on a scale from 0 to 1, were rescaled to a range of 0 to 10 by multiplying by ten. As a result, the odds ratios for the AI-ECG-AF model scores now reflect the change in odds associated with a 10% absolute increase in the AI-ECG-AF model score after adjusting for other clinical variables.

^a^ Logistic regression analysis 1 was adjusted for variables included in the POAF score.

^b^ Logistic regression analysis 2 was adjusted for variables extracted from the institutional cardiac surgery registry, as detailed in Supplementary Table S1, with variables selected using stepwise logistic regression with backward elimination.

OR: odds ratio; CI: confidence interval; POAF: postoperative atrial fibrillation.

**
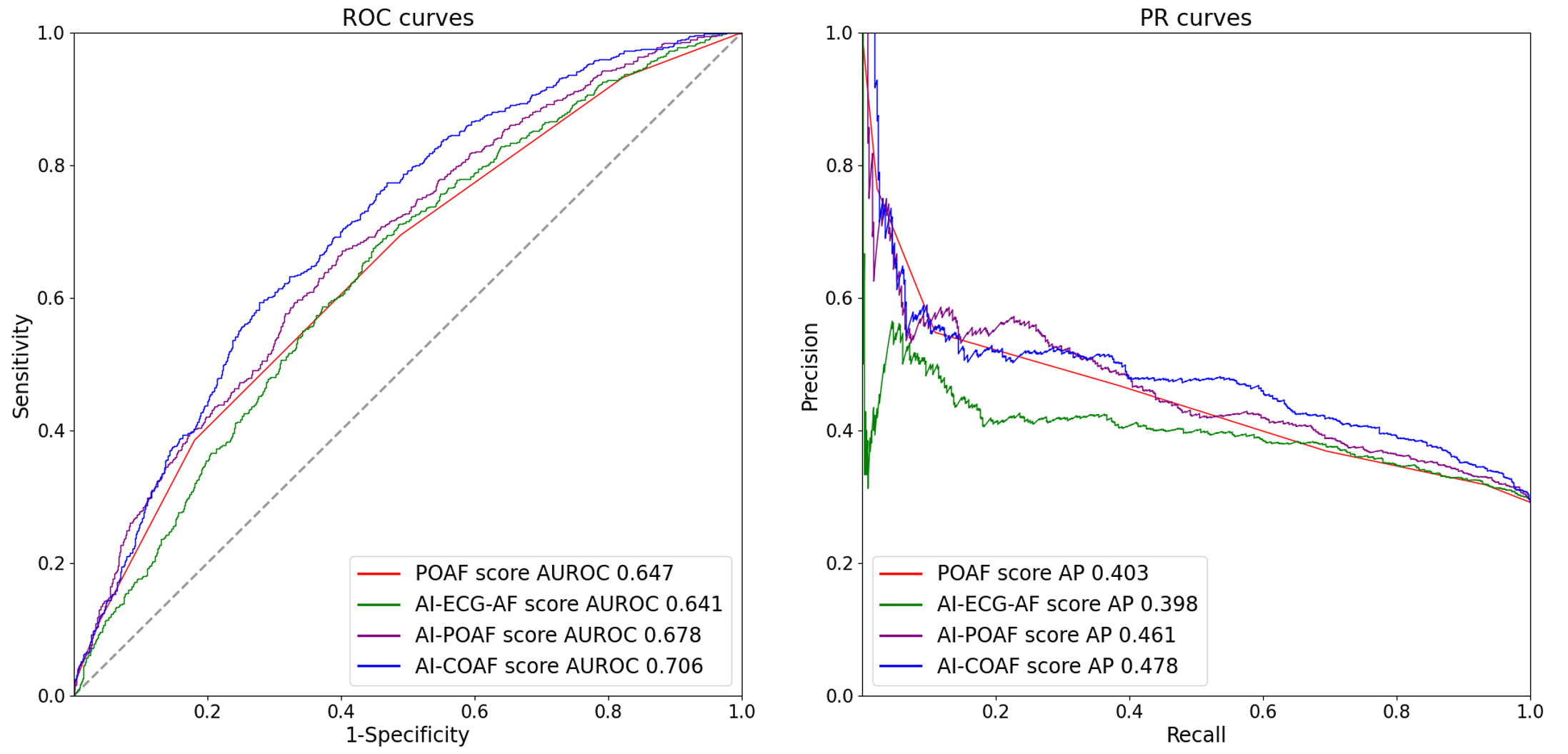
**

**Figure S4. ROC and PR curves of the models in the comparison dataset, based on a sensitivity analysis using a 7-day definition for postoperative AF.**

ROC: receiver operating characteristic; PR: precision-recall; AUROC: area under the receiver operating characteristics curve; AP: average precision; POAF: postoperative atrial fibrillation; AI: artificial intelligence; ECG: electrocardiogram; AF: atrial fibrillation; AI-COAF score: AI-enhanced post-cardiac-operative AF score.

**Supplementary Tables**

**Table S1. Preoperative clinical variables extracted from the institutional cardiac surgery registry.** Variables with missing rates exceeding 5% in the entire cohort were excluded, and the remaining missing data were handled using mean imputation.

| **Included variables** | **Excluded variables** |
| --- | --- |
| Age, Sex, Hypertension, Diabetes mellitus, Chronic kidney disease, Old cerebrovascular accident, Liver cirrhosis, Congestive heart failure, Chronic obstructive pulmonary disease, Old myocardial infarction, Recent myocardial infarction (3 months), Acute myocardial infarction (1 week), Emergency surgery, Height, Weight, Body surface area, Systolic blood pressure, Diastolic blood pressure, Left ventricular ejection fraction, White blood cells, Hemoglobin, Platelet, Sodium, Potassium, Blood urea nitrogen, Creatinine, Estimated glomerular filtration rate, Aspartate aminotransferase, Alanine aminotransferase, Albumin, Glucose, Prothrombin time, Partial thromboplastin time, Beta blockers, Calcium channel blockers, Angiotensin-converting enzyme inhibitors, Angiotensin receptor blockers, Statin, Oral nitrate, Diuretics, Warfarin, Heparinization, Intravenous nitrate, Non-vitamin K oral anticoagulant. | Calcium, Hemoglobin A1c, Creatinine kinase, Creatinine kinase-MB isoenzyme, Troponin T, Antithrombin III, Fibrinogen, C-reactive protein, Triiodothyronine, Free thyroxine, Thyroid stimulating hormone, Iron, Total Iron-Binding Capacity, Transferrin Saturation, Ferritin, Right ventricular pressure. |

**Table S2. Additional patient characteristics.** Preoperative clinical characteristics not included in Table 1 are presented.

| **Variable** | **Postoperative AF negative**  **(n = 3389)** | **Postoperative AF positive**  **(n = 1815)** | **P-Value** |
| --- | --- | --- | --- |
| **Age, years, median [IQR]** | 64 [54-71] | 69 [62-75] | <0.001 |
| **Hypertension, n (%)** | 1986 (58.6) | 1260 (69.4) | <0.001 |
| **Diabetes mellitus, n (%)** | 1149 (33.9) | 679 (37.4) | 0.012 |
| **Chronic kidney disease, n (%)** | 467 (13.8) | 392 (21.6) | <0.001 |
| **Old cerebrovascular accident, n (%)** | 353 (10.4) | 194 (10.7) | 0.796 |
| **Liver cirrhosis, n (%)** | 55 (1.6) | 33 (1.8) | 0.683 |
| **Congestive heart failure, n (%)** | 429 (12.7) | 371 (20.4) | <0.001 |
| **Old myocardial infarction, n (%)** | 290 (8.6) | 149 (8.2) | 0.706 |
| **Recent myocardial infarction (3 months), n (%)** | 325 (9.6) | 131 (7.2) | 0.005 |
| **Acute myocardial infarction (1 week), n (%)** | 351 (10.4) | 188 (10.4) | 1.000 |
| **Height, cm, median [IQR]** | 165 [159-170] | 164 [157-170] | <0.001 |
| **Weight, kg, median [IQR]** | 66.0 [57.4-74.2] | 64.5 [56.3-72.6] | <0.001 |
| **Body surface area, m^2^, median [IQR]** | 1.74 [1.61-1.86] | 1.72 [1.58-1.84] | <0.001 |
| **Systolic blood pressure, mmHg, median [IQR]** | 125 [113-137] | 126 [114-138] | 0.119 |
| **Diastolic blood pressure, mmHg, median [IQR]** | 74 [67-80] | 70 [64-78] | <0.001 |
| **LVEF, %, median [IQR]** | 62 [49-70] | 61 [45-68] | <0.001 |
| **White blood cells, /**$\boldsymbol{\mu L}$ **, median [IQR]** | 6580 [5400-8080] | 6190 [5035-7600] | <0.001 |
| **Hemoglobin,** $\boldsymbol{g/dL}$**, median [IQR]** | 12.5 [10.7-13.8] | 12.3 [10.3-13.6] | <0.001 |
| **Platelet,** $\boldsymbol{\times}$**10^3^/**$\boldsymbol{\mu L}$**, median [IQR]** | 214 [172-262] | 198 [161-242] | <0.001 |
| **Sodium, mmol/L, median [IQR]** | 140 [138-141] | 140 [138-142] | 0.940 |
| **Potassium, mmol/L, median [IQR]** | 4.2 [3.9-4.5] | 4.2 [3.9-4.4] | 0.146 |
| **Blood urea nitrogen,** $\boldsymbol{mg/dL}$**, median [IQR]** | 17.1 [13.3-22.2] | 19.0 [14.3-24.3] | <0.001 |
| **Creatinine,** $\boldsymbol{mg/dL}$**, median [IQR]** | 0.87 [0.73-1.07] | 0.89 [0.74-1.17] | 0.003 |
| **eGFR, mL/min/1.73m^2^, median [IQR]** | 87 [69-98] | 83 [60-92] | <0.001 |
| **Aspartate aminotransferase, IU/L, median [IQR]** | 24 [19-32] | 25 [19-32] | 0.671 |
| **Alanine aminotransferase, IU/L, median [IQR]** | 22 [15-32] | 19 [13-27] | <0.001 |
| **Albumin,** $\boldsymbol{g/dL}$**, median [IQR]** | 4.1 [3.7-4.4] | 4.0 [3.6-4.3] | <0.001 |
| **Glucose,** $\boldsymbol{mg/dL}$**, median [IQR]** | 110 [95-137] | 109 [95-137] | 0.887 |
| **PT, seconds, median [IQR]** | 11.8 [11.2-12.5] | 11.8 [11.3-12.5] | 0.072 |
| **PTT, seconds, median [IQR]** | 32.6 [30.1-40.0] | 32.1 [29.4-37.3] | <0.001 |
| **Beta blockers, n (%)** | 1494 (44.1) | 969 (53.4) | <0.001 |
| **Calcium channel blockers, n (%)** | 1154 (34.1) | 730 (40.2) | <0.001 |
| **Angiotensin-converting enzyme inhibitors, n (%)** | 316 (9.3) | 153 (8.4) | 0.306 |
| **Angiotensin receptor blockers, n (%)** | 1245 (36.7) | 832 (45.8) | <0.001 |
| **Statin, n (%)** | 2249 (66.4) | 1310 (72.2) | <0.001 |
| **Oral nitrate, n (%)** | 486 (14.3) | 325 (17.9) | 0.001 |
| **Diuretics, n (%)** | 1420 (41.9) | 922 (50.8) | <0.001 |
| **Warfarin, n (%)** | 61 (1.8) | 55 (3.0) | 0.006 |
| **Heparinization, n (%)** | 1222 (36.1) | 660 (36.4) | 0.850 |
| **Intravenous nitrate, n (%)** | 522 (15.4) | 318 (17.5) | 0.052 |
| **Non-vitamin K oral anticoagulant, n (%)** | 39 (1.2) | 18 (1.0) | 0.700 |

LVEF: left ventricular ejection fraction; eGFR: estimated glomerular filtration ratel PT: prothrombin time; PTT: partial thromboplastin time.

**Table S3. Results of logistic regression 1.** Adjusted for variables included in the POAF score. To facilitate a more intuitive interpretation, the AI-ECG-AF model scores, originally on a scale from 0 to 1, were rescaled to a range of 0 to 10 by multiplying by ten. As a result, the odds ratios for AI-ECG-AF model score * 10 now reflect the change in odds associated with a 10% absolute increase in the AI-ECG-AF model score after adjusting for other clinical variables.

| **Variable** | **OR (95% CI)** | **P-Value** |
| --- | --- | --- |
| **Age category** |  |  |
| **< 60 years** | Reference |  |
| **60 to 69 years** | 2.187 (1.865 – 2.569) | <0.001 |
| **70 to 79 years** | 2.883 (2.444 – 3.404) | <0.001 |
| **≥ 80 years** | 4.028 (2.932 – 5.552) | <0.001 |
| **COPD** | 1.747 (1.332 – 2.296) | <0.001 |
| **eGFR < 15 mL/min** | 0.895 (0.701 – 1.139) | 0.370 |
| **Emergency surgery** | 0.735 (0.499 – 1.070) | 0.114 |
| **Preoperative IABP/ECMO/VAD** | 1.268 (0.734 – 2.168) | 0.389 |
| **LVEF < 30%** | 0.988 (0.784 – 1.242) | 0.917 |
| **Valve surgery** | 1.293 (1.141 – 1.465) | <0.001 |
| **AI-ECG-AF model score * 10** | 1.197 (1.169 – 1.226) | <0.001 |

POAF: postoperative atrial fibrillation; OR: odds ratio; CI: confidence interval; COPD: chronic obstructive pulmonary disease; eGFR: estimated glomerular filtration rate; IABP: intra-aortic balloon pump; ECMO: extracorporeal membrane oxygenation; VAD: ventricular assist device; LVEF: left ventricular ejection fraction; AI: artificial intelligence; ECG: electrocardiogram; AF: atrial fibrillation.

**Table S4. Results of logistic regression 2.** Adjusted for variables extracted from the institutional cardiac surgery registry, as detailed in Supplementary Table S1, with variables selected using stepwise logistic regression with backward elimination. To facilitate a more intuitive interpretation, the AI-ECG-AF model scores, originally on a scale from 0 to 1, were rescaled to a range of 0 to 10 by multiplying by ten. As a result, the odds ratios for AI-ECG-AF model score * 10 now reflect the change in odds associated with a 10% absolute increase in the AI-ECG-AF model score after adjusting for other clinical variables.

| **Variable** | **OR (95% CI)** | **P-Value** |
| --- | --- | --- |
| **Age, years** | 1.043 (1.036 – 1.052) | <0.001 |
| **Sex (male)** | 0.751 (0.640 – 0.880) | <0.001 |
| **Valve surgery** | 1.148 (0.982 – 1.342) | 0.083 |
| **Hypertension** | 1.238 (1.055 – 1.455) | 0.009 |
| **Chronic obstructive pulmonary disease** | 1.981 (1.473 – 2.669) | <0.001 |
| **Chronic kidney disease** | 1.892 (1.494 – 2.398) | <0.001 |
| **Old cerebrovascular accident** | 0.816 (0.659 – 1.006) | 0.059 |
| **Congestive heart failure** | 1.198 (1.003 – 1.429) | 0.046 |
| **Old myocardial infarction** | 0.728 (0.572 – 0.923) | 0.009 |
| **Recent myocardial infarction (3 months)** | 0.592 (0.457 – 0.764) | <0.001 |
| **Acute myocardial infarction (1 week)** | 0.827 (0.647 – 1.054) | 0.127 |
| **Beta blockers** | 1.223 (1.070 – 1.398) | 0.003 |
| **Calcium channel blockers** | 1.118 (0.967 – 1.293) | 0.133 |
| **Angiotensin receptor blockers** | 1.110 (0.966 – 1.274) | 0.140 |
| **Warfarin** | 2.148 (1.400 – 3.295) | <0.001 |
| **Heparinization** | 1.167 (0.959 – 1.421) | 0.124 |
| **Intravenous nitrate** | 1.280 (1.047 – 1.565) | 0.016 |
| **Non-vitamin K oral anticoagulant** | 0.595 (0.317 – 1.082) | 0.096 |
| **Weight, kg** | 1.007 (1.000 – 1.013) | 0.041 |
| **Diastolic blood pressure, mmHg** | 0.986 (0.980 – 0.992) | <0.001 |
| **White blood cells,** $\boldsymbol{\times}$**10^2^/μL** | 0.994 (0.991 – 0.997) | <0.001 |
| **Hemoglobin, g/dL** | 1.094 (1.046 – 1.144) | <0.001 |
| **Platelet,** $\boldsymbol{\times}$**10^5^/μL** | 0.855 (0.783 – 0.934) | <0.001 |
| **Potassium, mmol/L** | 1.136 (0.969 – 1.331) | 0.115 |
| **Blood urea nitrogen, mg/dL** | 1.007 (1.001 – 1.013) | 0.024 |
| **Creatinine, mg/dL** | 1.059 (0.988 – 1.134) | 0.101 |
| **Estimated glomerular filtration rate, mL/min/1.73m^2^** | 1.013 (1.009 – 1.018) | <0.001 |
| **Albumin, g/dL** | 0.781 (0.668 – 0.912) | 0.002 |
| **Prothrombin time, seconds** | 0.996 (0.993 – 0.998) | <0.001 |
| **Partial thromboplastin time, seconds** | 0.989 (0.984 – 0.995) | <0.001 |
| **AI-ECG-AF score * 10** | 1.209 (1.178 – 1.242) | <0.001 |

POAF: postoperative atrial fibrillation; OR: odds ratio; CI: confidence interval; COPD: chronic obstructive pulmonary disease; eGFR: estimated glomerular filtration rate; IABP: intra-aortic balloon pump; ECMO: extracorporeal membrane oxygenation; VAD: ventricular assist device; LVEF: left ventricular ejection fraction; AI: artificial intelligence; ECG: electrocardiogram; AF: atrial fibrillation.
